# Changes of bioactive compounds in barley industry by‐products during submerged and solid state fermentation with antimicrobial *Pediococcus acidilactici* strain LUHS29

**DOI:** 10.1002/fsn3.1311

**Published:** 2019-12-13

**Authors:** Elena Bartkiene, Erika Mozuriene, Vita Lele, Egle Zokaityte, Romas Gruzauskas, Ida Jakobsone, Grazina Juodeikiene, Romas Ruibys, Vadims Bartkevics

**Affiliations:** ^1^ Lithuanian University of Health Sciences Kaunas Lithuania; ^2^ Kaunas University of Technology Kaunas Lithuania; ^3^ Centre of Food Chemistry University of Latvia Riga Latvia; ^4^ Institute of Food Safety Animal Health and Environment Riga Latvia; ^5^ Institute of Agricultural and Food Sciences Agriculture Academy Vytautas Magnus University Kaunas Lithuania

**Keywords:** barley, bioactive compounds, by‐products, lactic acid bacteria, *Pediococcus*

## Abstract

In this study, changes of bioactive compounds (crude protein (CP), crude fat (CF), dietary fiber (DF), fatty acids (FAs), free amino acids (FAAs), phenolic compounds (PCs), biogenic amines (BAs), lignans, and alkylresorcinols) in barley industry by‐products (BB) during submerged and solid state fermentation (SSF) with *Pediococcus acidilactici* were analyzed. It was established that both fermentation conditions reduce the CP and CF content in BB (by 25.8% and 35.9%, respectively) and increase DF content (on average by 25.0%). Fermentation increases the oleic, arachidic, eicosadienoic, behenic, and lignoceric FA in BB samples. The highest total BA content was found in untreated samples (290.6 mg/kg). Solid state fermentation increased the content of the alkylresorcinol C19:0. Finally, collecting data about the changes of these compounds during technological processes is very important, because according to the specific compounds formed during fermentation, further recommendations for by‐product valorization and uses in food, pharmaceutical, or feed industries can be suggested.

## Graphical abstract

The submerged and solid state fermentation with *Pediococcus acidilactici* change bioactive compounds content in barley industry by‐products. The changes of bioactive compounds during technological processes are very important, because the specific and useful compounds formed during fermentation. Fermented by‐product can be suggested for valorization and uses in food, pharmaceutical, or feed industries.

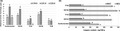

## 1 INTRODUCTION

The food processing industry in most countries across the world generates a huge quantity of by‐products, which have limited use and create considerable environmental pollution (Radenkovs, Juhnevica‐Radenkova, Górnaś, & Seglina, 2018). This waste causes a serious disposal problem for the environment. New approaches to use of these by‐products have become a very important issue, because most of them are an excellent source of various bioactive compounds: polyphenols, flavonoids, caffeine, carotenoids, creatine, polysaccharides, etc., which are beneficial for human, as well as animal, health (Wang et al., 2018). Cereal bran is regarded as an unavoidable by‐product of the milling industry with little commercial value, and so far, its main use is as a supplement for animal feed (Prückler et al., 2015). One of the major challenges is finding efficient technologies for valorization of the by‐products in an environmentally friendly manner. A way to add to the functionality of cereal by‐products is fermentation using lactic acid bacteria (LAB). Our previous study showed that fermentation with selected technological strains could be a promising solution for cereal by‐product valorization (Bartkiene et al., 2017). The use of LAB starter cultures with known characteristics offers a promising tool for in situ product enhancement, innovation, and diversification (Peyer, Zannini, & Arendt, 2016). An important characteristic of LAB is the bioconversion and biosynthesis of certain compounds that have a positive influence on human/animal health, as well as the potential use of LAB to enrich nutritional compounds and degrade the toxic components in foods (Wang et al., 2018). Fermentation with LAB has been proven as a very feasible option to enhance the technological, sensory, nutritional, and functional features of cereal industry by‐products. Through an increase of mineral, phenolic acid, amino acid, and vitamin bioavailability, as well as protein digestibility, a decrease in alkylresorcinol content, and the degradation of antinutritional compounds, fermentation can lead to improved nutritional quality of the matrix. In some cases, more compelling benefits have been discovered, such as the synthesis of bioactive compounds acting as antimicrobial, antitumoral, and antioxidant agents (Zhao, Guo, & Zhu, 2017). However, it must be taken into account that some decarboxylation reactions can lead to the formation of biogenic amines (BAs), which are harmful for human health (Renes, Ladero, Tornadijo, & Fresno, 2019).

The fermentation process can be performed under solid state (SSF), submerged (SMF), or liquid conditions, according to the specific product. Solid state fermentation, the growth of microorganisms on an adequately moistened nonsoluble medium in the absence or near absence of free‐moving water and air, is an area of interest to add value to by‐products using an inexpensive process (Ozmihci, 2017). The SSF process consists of maintaining conditions such as humidity and temperature closer to natural ones, without the need for drastic corrections in the substrate for fungus growth (Wang et al., 2018). For this reason, SSF has many advantages over SMF. From this point of view, SSF is presented as a promising technology for by‐product valorization through bioconversion into high value‐added products. Bran fermentation increases the content and bioavailability of several functional compounds, total free phenols, and soluble fiber (Manini et al., 2014).

Most of the studies, about the cereal by‐products valorization, are focused on the main nutritional compound changes (carbohydrates, protein, and fat profiles); however, studies about the changes of BAs, lignans, and alkylresorcinols during the cereal technological processes are scarce. However, abovementioned compounds can possess strong (desirable, as well as undesirable) physiological activities in vivo. The novelty of this study is based on possible cereal by‐products valorization by applying more sustainable technology—SSF. Also, versatile results, about the both desirable and undesirable compounds formation, in cereal by‐products can be useful for the further, such type of products industrialization in food/feed/nutraceutical sectors.

In this study, changes of the bioactive compounds (fatty acid and amino acid profile, phenolic compounds, BAs, lignans, and alkylresorcinols) in barley industry by‐products during SMF and SSF with *Pediococcus acidilactici* strain LUHS29, which shows antimicrobial properties, were analyzed.

## 2 MATERIALS AND METHODS

### 2.1 Barley crop industry by‐products and their fermentation with *P. acidilactici* strain LUHS29

The grounded barley by‐products were obtained from local mill (Ustukiu malunas Ltd., Pasvalys, Lithuania) in 2018. Cereal by‐products samples were collected according to standard procedures described in ISO 24333:2009 (Cereals and cereal products—Sampling, ISO, 2009:^2009^). Samples were collected during barley milling process and stored in the dark at ambient temperature until experiment. The *P. acidilactici* strain LUHS29, previously isolated from spontaneous fermented cereals and which showed broad antimicrobial activity against opportunistic and pathogenic strains (Bartkiene et al., 2019), was stored at −80°C and cultured at 30°C for 48 hr in MRS broth (CM0359, Oxoid Ltd) with the addition of 40 mmol/L fructose and 20 mmol/L maltose prior to use. The fermentation of barley by‐products was performed with a multiplied *P. acidilactici* strain (3% by volume of the pure *P. acidilactici* strain, diluted in MRS broth added to cereal/water mass). Three parallel replicates of fermented samples were prepared, and each fermented sample analysis was repeated three times. The water content of the end‐product for SSF was 450 g/kg; for SMF, it was 650 g/kg. Fermentation was carried out for 72 hr at 32 ± 2°C. Unfermented barley by‐products were used as a control.

### 2.2 Methods for evaluating the content of crude protein, fat, ash, fiber, and their fractions (NDF, ADF, and ADL) in barley crop industry by‐products

Ash content was determined by calcinations at 900°C (ICC^1990^/1990:, 1990. Determination of ash in cereals and cereal products). Nitrogen content was determined using Kjeldahl method with a factor of 5.7 to determine protein content (ICC^2001^/2001:, 2001. Determination of crude protein in cereals and cereal products for food and for feed). The total lipid content was determined by extraction in the Soxhlet apparatus (“Boeco”) with hexane technical grade (Fisher Scientific) (ICC^1984^:, 1984. Cereals and cereal products—Determination of total fat content). Neutral detergent fiber (NDF) and acid detergent fiber (ADF) were analyzed according to Van Soest, Robertson, and Lewis (1991). Analysis of NDF, ADF, and acid detergent lignin (ADL) was carried out using an ANKOM 200 Fiber Analyzer Unit (ANKOM Technology Corp.). Neutral detergent fiber was assayed with the use of alpha amylase and sodium sulfite in the NDF. Both NDF and ADF were expressed without residual ash (AOAC, 2000).

### 2.3 Evaluation of the fatty acid (FA) and free amino acid (FAA) profile in fermented and nonfermented barley by‐products

The FA profile of the barley fat fraction was determined using gas chromatography–flame ionization detection (GC‐FID; Agilent 6890N Gas Chromatograph, Agilent Technologies), according to the procedure described by Bartkiene, Bartkevics, Starkute, et al. (2016).

FAA was extracted using 0.1 M HCl. The extracts were processed by ion‐exchange solid phase extraction and chloroformate derivatization using EZ:faast^®^ technology (Phenomenex) and then analyzed by GC‐FID. Standard solutions of the amino acids (AA) alanine (Ala), glycine (Gly), valine (Val), leucine (Leu), isoleucine (Ile), threonine (Thr), serine (Ser), proline (Pro), asparagine (Asp), methionine (Met), glutamine (Glu), phenylalanine (Phe), lysine (Lys), histidine (His), and tyrosine (Tyr) were analyzed, in addition to the internal standard (Nval). All eluting and derivatization agents were provided in an inclusive kit (EZ‐Fast Amino acid Analysis Kit for protein hydrolysates by GC‐FID or GC‐NPD). The derivatized AA were analyzed using a GC‐FID instrument (Agilent 6890N) equipped with an auto‐sampler (Agilent 7683 Series) according to the method described by Bartkiene, Bartkevics, Rusko, et al. (2016).

### 2.4 Evaluation of phenolic acids (PAs) in barley by‐products

Stock solutions of the standard acids were prepared at a concentration of 1.0 g/100 ml in pure methanol. The working solutions of samples were prepared at a concentration of 1.0 g/100 ml in methanol. The acid mixtures were separated on a Shimadzu LC‐9A model HPLC equipped with a manual injector, a programmable wavelength photodiode array UV detector (200–400 nm), and column packing with modified silica gel (C_18_ column). Extraction and evaluation of PAs were performed according to method described by Tüzen and Özdemir (2003).

### 2.5 Evaluation of BA content in barley by‐products

Sample preparation and determination of BAs in barley by‐products were performed according to the method of Ben‐Gigirey, Sousa, Villa, and Barros‐Velazquez (1999), with some modifications described by Bartkiene, Bartkevics, Rusko, et al. (2016). Perchloric acid (0.4 M, 10 ml) containing a known amount of 1,7‐diaminoheptane used as an internal standard was added to 3 g of sample, and the mixture was homogenized with Ultra‐Turrax (IKA Labortechnik) and centrifuged (3,000 *g*, 4°C, 10 min). The residue was extracted again with an equal volume of 0.4 M perchloric acid. Both supernatants were combined, and the final volume was adjusted to 30 ml with 0.4 M perchloric acid. The extract was filtered through Whatman paper No. 1. One milliliter of extract or standard solution was mixed with 200 μL of 2 M sodium hydroxide and 300 μL of saturated sodium bicarbonate. A 5‐(dimethylamino)naphthalene‐1‐sulfonyl chloride (dansyl chloride reagent) (10 mg/ml, 2 ml) prepared in acetone was added to the mixture and incubated at 40°C for 45 min. Residual dansyl chloride was removed by the addition of 100 μL of 25% ammonium hydroxide. After incubation at room temperature for 30 min, the mixture was adjusted to 5 ml with acetonitrile. Finally, the mixture was centrifuged (3,000 *g*, 5 min), and the supernatant was filtered through 0.2‐μm filters (Millipore Co.) and was kept at −25°C until HPLC analysis. An Agilent 1200 HPLC (Agilent) equipped with DAD detector and Chemstation LC software was employed. A Chromolith C18 HPLC column (100 mm·4.6 mm·4 μm, Merck KGaA/EMD Chemicals) was used. Ammonium acetate (0.1 M) and acetonitrile were used as the mobile phases by a flow rate of 0.45 ml/min. The sample volume injected was 10 μL, and the amines were monitored by 254 nm. The detection limits for standard amine solutions were approximately 0.1 mg/kg.

### 2.6 Analysis of alkylresorcinol and lignan concentration in barley crop industry by‐products

For the determination of alkylresorcinols, barley by‐product samples were placed in 50 ml tubes and extracted by continuous shaking for 24 hr at room temperature (20°C, or rotation), with 40 ml of ethyl acetate containing 0.5 mg (or 0.500 μg/mL → 200 μL) of methyl behenate internal standard and centrifuged for 10 min at 1,500 g (~6,000 rpm, *r* = 4 cm). Portions (4 ml) of the extract were transferred to 5 ml test tubes and then dried by evaporation *in vacuo* using a centrifuge evaporator for 40 min. Ethyl acetate (200 μL) was added, and the samples were mixed and filtered through 0.45‐μm filters (GHP Acrodisc) then transferred to GC vials for analysis (Annica, Andersson, Åman, Wandel, & Frølich, 2010). The GC/MS analysis was performed according to method described by Bartkiene et al. (2015) on an HP 5890 II gas chromatograph coupled to a TRIO‐1000 mass spectrometer with a LAB‐BASE data system (version R2.10; Fision Instruments).

For the determination of lignans in barley by‐product samples, extraction was performed according to the methods described by Krajčová, Schulzová, Hajšlová, and Bjelková (2009). The analysis was performed according to the method described by Bartkiene et al. (2017). Defatted (*n*‐hexane, 2 hr at 60°C) samples (0.5 g) were mixed with 12 ml of 0.3 M NaOH in methanol/water (70/30, v/v) and incubated for 1 hr at 60°C. The hydrolysate was neutralized with glacial acetic acid and centrifuged (10 min, 3,000 *g*). An aliquot of 0.5 ml was evaporated to dryness, dissolved in 3 ml of sodium acetate buffer (0.1 M, pH 5.0) with 400 μL of β‐glucuronidase/sulfatase enzyme (from *Helix pomatia*), and incubated overnight at 37°C. The enzymatic hydrolysate was extracted twice with 3 ml of diethyl ether, and the two organic phases were combined and evaporated to dryness (under nitrogen with gentle heating, max 55°C, on a water bath). The dried sample was dissolved in 0.5 ml of methanol. The β‐glucuronidase/sulfatase enzymes (type H2, from *H. pomatia*, 11,400 U/mL of β‐glucuronidase and 3,290 U/mL of sulfatase) were purchased from Sigma (Sigma‐Aldrich). The HPLC‐MS/MS analysis was performed on an Alliance Module 2695 (Waters) with separation on a Discovery C18 column (50 mm × 3.0 mm i.d., 5 μm) (Supelco). The mobile phase consisted of 0.5% acetic acid in water and 0.5% acetic acid in methanol. Detection was performed with a Quattro Premier XE (Waters) employing an electrospray ionization source. For detection, the characteristic precursor and product ions were combined: secoisolariciresinol (*m/z* 361.2 > *m/z* 165.0) and matairesinol (*m/z* 357.1 > *m/z* 82.7).

### 2.7 Statistical analysis

The results were expressed as the mean value of measurements ± standard deviation. Three parallel replicates of fermented samples were prepared, and each fermented sample analysis was repeated three times. In order to evaluate the effects of the different fermentation conditions (SSF and SMF, as well as different durations of fermentation), the data were analyzed by the analysis of variance (IBM SPSS Statistics, ver. 22). Results were recognized as statistically significant at *p* ≤ .05.

## 3 RESULTS AND DISCUSSION

### 3.1 Content of crude protein, fat, fiber, and their fractions (NDF, ADF, and ADL) in barley industry by‐products

The chemical composition of the untreated and fermented barley by‐products (BB) is given in Table 1. In all cases, fermentation reduced the crude protein content in BB compared with untreated samples: A crude protein content lower by 29.4%, 26.8%, and 21.2% was established in SSF samples after 24, 48, and 72 hr, respectively. As well as that, a crude protein content lower by 26.9%, 26.7%, and 25.2% was found in SMF samples after 24, 48, and 72 hr, respectively. Lactic acid fermentation significantly reduces the protein content, and a slight decrease in protein content could be explained by the capability of LAB to degrade proteins in fermentable substrates (Gardini, Özogul, Suzzi, Tabanelli, & Özogul, 2016), as LAB are capable of producing proteinases. However, comparing SSF samples, the greatest decrease of crude protein was found after 24 hr of fermentation (compared with untreated samples, decreased by 29.4%); comparing crude protein content after 48 and 72 hr of fermentation with protein content after 24 hr of SSF, it was higher in samples fermented for longer by 0.44% and 1.38%, respectively. It could be that the main proteolytic enzyme activity of LAB occurred during the 24 hr and, after that, activity was reduced by inhibiting LAB cells; the small increase in crude protein can be explained by the presence of bacterial cell proteins in the SSF substrate.

**Table 1:** Changes in the content of crude protein, crude fat, crude fiber, and their fractions (NDF, ADF, and ADL) in barley by‐products (BB) fermented with a *Pediococcus acidilactici* strain under submerged (SMF) and solid state (SSF) fermentation conditions

Parameters	Nonfermented samples	After 24 hr		After 48 hr		After 72 hr	
		SSF	SMF	SSF	SMF	SSF	SMF
Crude protein	16.73 ± 0.21^c^	11.81 ± 0.08^a^	12.23 ± 0.14^b^	12.25 ± 0.09^a^	12.26 ± 0.51^c^	13.19 ± 0.32^c^	12.52 ± 0.58^c^
Crude fat	4.20 ± 0.14^b^	2.54 ± 0.10^b^	2.70 ± 0.13^b^	2.66 ± 0.07^a^	2.83 ± 0.16^b^	2.54 ± 0.15^a^	2.88 ± 0.11^a^
Crude fiber	12.34 ± 0.16^b^	15.14 ± 0.07^a^	14.13 ± 0.09^b^	14.53 ± 0.14^b^	15.73 ± 0.68^c^	16.85 ± 1.36^e^	16.16 ± 1.36^e^
NDF	4.10 ± 0.09^a^	5.15 ± 0.11^b^	5.57 ± 0.16^c^	5.39 ± 0.11^b^	4.73 ± 0.28^c^	5.17 ± 0.28^b^	5.51 ± 0.74^d^
ADF	17.58 ± 0.11^b^	20.10 ± 0.14^c^	21.87 ± 0.21^c^	21.54 ± 0.36^c^	21.18 ± 0.31^c^	21.33 ± 1.25^b^	21.57 ± 1.34^e^
ADL	37.72 ± 0.17^b^	45.77 ± 0.25^c^	42.25 ± 1.63^d^	48.10 ± 1.85^e^	44.43 ± 0.27^b^	45.71 ± 2.69^e^	41.59 ± 2.30^e^

Note

Data are the mean ± *SD* (*n* = 3).

Abbreviations: ADF, acid detergent fiber; ADL, acid detergent lignin; NDF, neutral detergent fiber; SMF, submerged fermentation; SSF, solid state fermentation.

^a–e^Mean values with different letters are significantly different when *p* ≤ .05.

Also, in fermented BB samples, crude fat content was reduced compared with control samples in SSF samples by 39.5%, 36.7%, and 39.5% after 24, 48, and 72 hr, respectively, and in SMF samples by 35.7%, 32.6% and 31.4%, respectively. Lactic acid bacteria can contribute to the formation of free FAs, which can be precursors of characteristic aroma compounds and lactones in some fermented products (Gardini et al., 2016). However, LAB are generally advantageous for use as a starter culture in conditions of low lipolytic activity (Yalçınkaya & Kılıç, 2019). Opposite to the changes observed for crude protein and fat, an increase of crude fiber was established in fermented samples after 24, 48, and 72 hr in SSF samples by 22.7%, 17.8%, and 36.6%, respectively, and in SMF samples by 14.5%, 27.5%, and 31.0%, respectively. Lactic acid bacteria increase the concentrations of NDF, ADF, ADL, and hemicelluloses in fermentable substrates (Wang et al., 2018). In this study, the same tendencies were established, as NDF, ADF, and ADL fractions were higher in fermented BB samples than in untreated samples. Compared with untreated BB, in SSF and SMF samples, NDF, ADF, and ADL were found to be higher by 27.8% and 28.5%, 19.4% and 22.5%, and 23.4% and 13.5%, respectively. The experimental results are in agreement with Li, Zhou, Zi, and Cai (2017) who determined that LAB can improve fermentation quality, chemical composition, and bioavailability (inhibiting protein degradation and promoting fiber degradation), thus having great potential as an additive for bran fermentation.

### 3.2 FA composition of the fermented barley by‐products

The FA profile of the BB is given in Table 2. Under both fermentation conditions (SSF and SMF), increases of the oleic, arachidic, eicosadienoic, behenic, and lignoceric acid content were established. Different tendencies of the other FAs identified were found. Compared with untreated BB samples, the myristic acid content was 30.8% higher in 24 hr SSF samples; however, after 48 hr of SSF, it was lower by 15.4%, and after 72 hr, it was similar to that in untreated samples. In all cases, a lower myristic acid content was found in SMF samples compared with untreated samples (on average 19.2% lower). Comparing palmitic acid content, after 24 and 48 hr of SSF, it was 14.4% and 11.7% higher, respectively, than in untreated samples; however, after 72 hr of SSF, it was similar to that in untreated samples. In all cases, a lower palmitic acid content was found in SMF samples compared with untreated samples (on average 7.5% lower). By increasing fermentation duration, the hexadecanoic FA content was increased by 26.7% and 33.3% in SSF and SMF samples after 72 hr of fermentation, respectively. Also, in most of the fermented samples (except 24 hr SMF), a higher stearic FA content was found. Different tendencies were found for vaccenic FA, and the highest content was found in 48 hr SSF samples (0.42 ± 0.01%). In SMF samples, the linoleic FA content was similar to that in untreated samples; however, a decrease of linoleic acid content was established in SSF samples (on average, a 17.3% decrease). Linolenic FA decreased in all fermented samples, and the lowest content was found in 48 hr SSF samples (3.97 ± 0.05%). No significant changes of eicosadienoic FA after fermentation were established. Arachidonic FA was not determined in untreated BB samples; however, in 24 and 48 hr SSF and SMF samples, its content ranged from 0.1% to 0.57% (in 24 and 48 hr SSF samples, respectively). But after 72 hr of SSF, arachidonic FA was not determined. In most of the fermented samples, a higher eicosapentaenoic FA content was established compared with untreated samples; the exceptions were 48 and 72 hr SMF samples in which the eicosapentaenoic FA content was similar to that in untreated samples. The highest increase of erucic FA was found in 24 hr SMF samples compared with untreated samples (increased by 71.4%). Also, in most of the fermented samples (except 48 hr SMF), the docosahexaenoic FA content was increased (on average by 50.7%). Different tendencies were found for nervonic FA changes, as after 48 and 72 hr of SSF, nervonic FA content in BB samples increased by 93.3% and 100.0%, respectively. No trans‐isomers were established in BB samples. Prückler et al. (2015) provided the FA necessary for LAB growth. Moreover, Pontonio, Dingeo, Gobbetti, and Rizzello (2019) determined that fermentation allows a marked decrease of lipase activity, stabilizing the matrix by preventing oxidative processes, and can be proposed as a valuable alternative to decreasing lipase activity. Chemical compounds (e.g., organic acids, FAs) present in cereals, or released from LAB as intermediate compounds during fermentation, can be channelled into different metabolic pathways that ultimately lead to specific organoleptic compounds (Gänzle, Vermeulen, & Vogel, 2007).

**Table 2:** Changes of fatty acid composition in barley by‐products (BB) fermented with a *Pediococcus acidilactici* strain under submerged (SMF) and solid state (SSF) fermentation conditions

Acid	Nonfermented samples	After 24 hr		After 48 hr		After 72 hr	
		SSF	SMF	SSF	SMF	SSF	SMF
Myristic	0.26 ± 0.02^a^	0.34 ± 0.01^a^	0.19 ± 0.01	0.26 ± 0.01^a^	0.22 ± 0.01^a^	0.25 ± 0.01^a^	0.22 ± 0.01^a^
Palmitic	20.36 ± 0.05^b^	23.30 ± 0.02^a^	18.75 ± 0.03^a^	22.74 ± 0.02^c^	18.78 ± 0.03^a^	20.45 ± 0.04^b^	18.99 ± 0.02^a^
Hexadecanoic	0.30 ± 0.04^a^	0.22 ± 0.01^a^	0.17 ± 0.01^a^	0.33 ± 0.01^a^	0.13 ± 0.01^a^	0.38 ± 0.01^a^	0.40 ± 0.01^a^
Stearic	1.40 ± 0.03^a^	1.94 ± 0.02^a^	1.40 ± 0.02^a^	2.19 ± 0.04	1.54 ± 0.03^a^	1.76 ± 0.04^b^	2.09 ± 0.01^a^
Oleic	14.85 ± 0.02^a^	20.42 ± 0.04^a^	16.50 ± 0.04^a^	21.53 ± 0.03^a^	15.21 ± 0.02^a^	21.90 ± 0.05^b^	16.61 ± 0.04^b^
Vaccenic	0.28 ± 0.03^a^	0.37 ± 0.01^a^	0.30 ± 0.02^a^	0.42 ± 0.01^a^	0.26 ± 0.02^a^	0.33 ± 0.04^b^	0.29 ± 0.02^a^
Linoleic	54.89 ± 0.02^a^	45.49 ± 0.03^a^	54.96 ± 0.04^b^	44.46 ± 0.05^c^	56.24 ± 0.04^b^	46.20 ± 0.03^a^	53.41 ± 0.02^a^
Linolenic	5.28 ± 0.05^b^	4.09 ± 0.06^b^	4.63 ± 0.07^b^	3.97 ± 0.05^b^	4.84 ± 0.03^b^	4.37 ± 0.01^a^	4.84 ± 0.02^a^
Arachidic	0.22 ± 0.01^a^	0.36 ± 0.01^a^	0.30 ± 0.03^a^	0.35 ± 0.04^a^	0.28 ± 0.03^a^	0.34 ± 0.04^a^	0.32 ± 0.05^a^
Eicosenoic	0.69 ± 0.01^a^	0.85 ± 0.02^a^	0.75 ± 0.05^b^	0.87 ± 0.03^a^	0.74 ± 0.04^a^	0.85 ± 0.01^a^	0.79 ± 0.02^a^
Eicosadienoic	0.11 ± 0.01^a^	0.14 ± 0.02^a^	0.13 ± 0.01^a^	0.14 ± 0.02^a^	0.11 ± 0.01^a^	0.14 ± 0.02^a^	0.11 ± 0.02^a^
Arachidonic	–	0.10 ± 0.01^a^	0.12 ± 0.01^a^	0.57 ± 0.03^a^	0.09 ± 0.01^a^	–	0.10 ± 0.01^a^
Eicosapentaenoic	0.17 ± 0.02^a^	0.36 ± 0.02^a^	0.26 ± 0.03^a^	0.35 ± 0.04^b^	0.17 ± 0.01^a^	0.34 ± 0.02^a^	0.19 ± 0.01^a^
Behenic	0.23 ± 0.03^a^	0.44 ± 0.02^a^	0.31 ± 0.05^b^	0.36 ± 0.02^a^	0.31 ± 0.02^a^	0.36 ± 0.01^a^	0.32 ± 0.01^a^
Erucic	0.14 ± 0.01^a^	0.17 ± 0.01^a^	0.24 ± 0.01^a^	0.18 ± 0.02^a^	0.19 ± 0.01^a^	0.17 ± 0.02^a^	0.15 ± 0.01^a^
Docosahexaenoic	0.15 ± 0.01^a^	0.19 ± 0.02^a^	0.20 ± 0.03^a^	0.24 ± 0.02^a^	0.15 ± 0.02^a^	0.27 ± 0.03^a^	0.23 ± 0.03^b^
Lignoceric	0.16 ± 0.01^a^	0.27 ± 0.02^a^	0.28 ± 0.02^a^	0.25 ± 0.02^a^	0.31 ± 0.03^b^	0.68 ± 0.03^b^	0.24 ± 0.02^a^
Nervonic	0.15 ± 0.01^a^	0.14 ± 0.01^a^	0.12 ± 0.01^a^	0.29 ± 0.02^b^	0.13 ± 0.02^a^	0.30 ± 0.03^b^	0.13 ± 0.01^a^
*Nonidentified*	0.36 ± 0.02^a^	0.81 ± 0.02^a^	0.38 ± 0.02^a^	0.50 ± 0.02^b^	0.30 ± 0.01^a^	0.91 ± 0.03^a^	0.57 ± 0.02^a^
*trans‐isomer*	–	–	–	–	–	–	–
SFA	22.62 ± 0.05^b^	26.64 ± 2.68^c^	21.20 ± 3.01^c^	26.14 ± 3.41^c^	21.44 ± 10.33^c^	23.43 ± 3.68^c^	22.18 ± 3.28^c^
MUFA	16.36 ± 0.03^b^	22.17 ± 2.19^c^	17.98 ± 2.25^c^	23.62 ± 2.14^c^	16.60 ± 2.55^b^	23.93 ± 2.18^c^	18.35 ± 6.34^c^
PUFA	60.60 ± 8.25^d^	50.37 ± 3.54^d^	60.29 ± 10.51^d^	49.28 ± 5.12^d^	61.60 ± 15.69^d^	51.27 ± 9.52^c^	58.88 ± 10.36^d^

Note

Data are the mean ± *SD* (*n* = 3).

Abbreviations: MFA, monounsaturated fatty acids; n‐3, omega‐3 fatty acid; n‐6, omega‐6 fatty acid; PUFA, polyunsaturated fatty acids; SFA, saturated fatty acids; SMF, submerged fermentation; SSF, solid state fermentation; TI, thrombogenic index.

^a–e^Mean values with different letters are significantly different when *p* ≤ .05.

### 3.3 FAA profile of the barley by‐products

The FAA profile of the untreated and fermented BB is given in Table 3. In all cases, compared with untreated BB samples, FAAs in fermented BB were reduced; however, most of the changes depended on the fermentation duration. In 24, 48, and 72 hr SSF samples, aspartic acid was reduced by 26.7%, 23.4%, and 13.8%, respectively, and in 24, 48, and 72 hr SMF samples, it was reduced by 29.7%, 28.7%, and 34.6%, respectively, compared with untreated samples. On average, the threonine content was lower by 24.4% and 33.1% in SSF and SMF samples, respectively. Comparing serine content, the greatest reduction was found in 72 hr SMF samples (reduced by 38.0%). Glutamic acid in SSF samples on average reduced by 18.2%, and in SMF samples by 24.5%. The proline content in fermented samples was influenced more by fermentation duration than by fermentation conditions, and on average was reduced after 24 hr by 20.8%, after 48 hr by 51.9%, and after 72 hr by 24.1%. On average, glycine content was 19.7% lower in fermented samples, with the greatest reduction (by 24.9%) in 72 hr SMF samples. In all cases, a greater reduction of alanine was found in SMF samples (after 24 hr – 4.9%, after 48 hr – 8.8%, after 72 hr – 15.1%) compared with SSF. Similar tendencies were found for valine changes, and the smallest reduction was established in SMF samples, with the greatest reduction after 72 hr of fermentation (reduced by 30.3%). Comparing the methionine content in untreated and fermented samples, a reduction by 10.5%, 25.6%, and 9.0% was established in SSF samples after 24, 48, and 72 hr of fermentation, respectively, and in SMF samples by 2.3%, 9.0%, and 36.8%, respectively. In all cases, a greater reduction of isoleucine was found in SMF samples compared with SSF samples (content lower after 24 hr by 5.4%, after 48 hr by 6.6%, and after 72 hr by 18.3%, compared with SSF samples). The same tendencies were found for leucine, as in all cases a lower leucine content was found in SMF samples, compared with SSF, with the greatest decrease in 72 hr SMF samples (reduced by 32.8%, compared with untreated samples). Also, a lower tyrosine content was found in SMF samples (after 24 hr – 21.0%, after 48 hr – 10.0%, after 72 hr – 10.4%), compared with SSF. In 24, 48, and 72 hr SSF samples, phenylamine was reduced by 24.1%, 28.2%, and 24.9%, respectively, and in 24, 48, and 72 hr SMF samples by 27.9%, 31.0%, and 35.3%, respectively, compared with untreated samples. Comparing histidine content, in SSF samples it was lower on average by 18.6% and in SMF on average by 24.6%, compared with untreated samples. Lysine in fermented samples was reduced on average by 40.8%, compared with untreated samples. In all cases, a greater reduction of arginine was found in SMF samples, compared with SSF samples (content lower after 24 hr by 8.6%, after 48 hr by 8.5%, and after 72 hr by 11.2%, compared with SSF samples). In this study, decreases were established in the total FAA content during fermentation, which was also reported by Khan et al. (2018). This decrease could be attributed to the consumption of FAAs during microbial metabolism and enzymatic conversion, and significant variation in regard to different FAAs throughout fermentation can be established (Khan et al., 2018). Arginine can be metabolized into ornithine and free ammonia through the arginine deiminase pathway during lactic acid fermentation whereas glutamine can be transformed to γ‐aminobutyric acid by glutamate decarboxylase produced by LAB, because groups of FAAs, such as branched‐chain (leucine, isoleucine, valine), aromatic (phenylalanine, pyramine), sulfuric (cystine), and acidic (asparagine), are converted into flavor compounds under the effect of aminotransferases generated by LAB so the overall levels tend to be lower. Moreover, threonine can be transformed into acetaldehyde under the catalysis of threonine aldolase, while glycine can be formed simultaneously, and a promotion in the concentration of glycine can be detectable in the later stages of fermentation (Wang et al., 2018).

**Table 3:** Changes in the amino acid profile of barley by‐products (BB) fermented with a *Pediococcus acidilactici* strain under submerged (SMF) and solid state (SSF) fermentation conditions

Amino acid	Nonfermented samples	After 24 hr		After 48 hr		After 72	
		SSF	SMF	SSF	SMF	SSF	SMF
Aspartic acid	9.45 ± 0.65^c^	6.93 ± 0.01^a^	6.64 ± 0.05^a^	7.24 ± 0.44^c^	6.74 ± 0.36^c^	8.15 ± 0.00^a^	6.18 ± 0.08^a^
Threonine	5.08 ± 0.06^a^	3.90 ± 0.02^a^	3.57 ± 0.08^a^	3.76 ± 0.05^a^	3.41 ± 0.07^a^	3.85 ± 0.04^a^	3.22 ± 0.03^a^
Serine	5.87 ± 0.40^c^	4.53 ± 0.04^a^	4.09 ± 0.07^a^	4.38 ± 0.05^a^	3.91 ± 0.12^b^	4.32 ± 0.10^b^	3.64 ± 0.09^b^
Glutamic acid	29.33 ± 0.89^c^	23.34 ± 0.19^b^	22.92 ± 0.28^b^	24.16 ± 0.84	22.92 ± 0.31^b^	24.58 ± 0.03^a^	21.70 ± 0.50^d^
Proline	11.47 ± 0.79^c^	9.10 ± 0.03^a^	9.05 ± 0.06^a^	9.74 ± 0.03^a^	9.30 ± 0.49^b^	8.63 ± 0.41^c^	8.79 ± 0.15^c^
Glycine	6.76 ± 0.07^a^	5.83 ± 0.05^a^	5.27 ± 0.39^b^	5.77 ± 0.04^a^	5.17 ± 0.22^b^	5.45 ± 0.04^a^	5.08 ± 0.11^c^
Alanine	6.27 ± 0.12^a^	5.06 ± 0.14^b^	4.81 ± 0.03^a^	5.13 ± 0.10^b^	4.68 ± 0.05^a^	5.24 ± 0.10^b^	4.45 ± 0.02^a^
Valine	6.87 ± 0.20^b^	5.42 ± 0.27^b^	5.28 ± 0.03^a^	5.52 ± 0.06^b^	5.14 ± 0.14^b^	5.55 ± 0.22^b^	4.79 ± 0.02^a^
Methionine	1.33 ± 0.01^a^	1.19 ± 0.29^b^	1.30 ± 0.17^b^	0.99 ± 0.06^b^	1.21 ± 0.16^b^	1.21 ± 0.52^c^	0.84 ± 0.22^c^
Isoleucine	4.91 ± 0.17^b^	3.87 ± 0.08^a^	3.66 ± 0.03^a^	3.91 ± 0.14^b^	3.65 ± 0.03^a^	4.10 ± 0.04^a^	3.35 ± 0.04^a^
Leucine	9.86 ± 0.21^b^	7.84 ± 0.16^b^	7.36 ± 0.03^a^	7.60 ± 0.26^c^	7.07 ± 0.06^a^	7.66 ± 0.23^c^	6.63 ± 0.06^a^
Tyrosine	3.47 ± 0.11^a^	3.05 ± 0.65^c^	2.41 ± 0.03^a^	2.51 ± 0.14^b^	2.26 ± 0.08^a^	2.49 ± 0.03^a^	2.23 ± 0.10^b^
Phenylamine	6.06 ± 0.21^b^	4.60 ± 0.05^a^	4.37 ± 0.08^a^	4.35 ± 0.20^b^	4.18 ± 0.03^a^	4.55 ± 0.14^b^	3.92 ± 0.05^a^
Histidine	4.14 ± 0.19^b^	3.38 ± 0.15^b^	3.09 ± 0.07^a^	3.55 ± 0.03^a^	3.06 ± 0.25^b^	3.17 ± 0.19^c^	3.22 ± 0.19^b^
Lysine	5.24 ± 0.12^a^	2.92 ± 0.09^a^	2.86 ± 0.08^a^	3.24 ± 0.11^a^	3.00 ± 0.09^a^	3.97 ± 0.06^a^	2.63 ± 0.08^b^
Arginine	9.27 ± 0.33^c^	6.51 ± 0.22^b^	5.95 ± 0.14^b^	6.00 ± 0.07^a^	5.49 ± 0.23^b^	5.74 ± 0.09^a^	5.10 ± 0.04^a^

Note

Data are the mean ± *SD* (*n* = 3).

Abbreviations: SMF, submerged fermentation; SSF, solid state fermentation.

^a–e^Mean values with different letters are significantly different when *p* ≤ .05.

### 3.4 Changes of PA content in barley by‐products

The PA content in untreated and fermented BB is shown in Table 4. Comparing vanillic acid content, after 24 hr of SSF it was 22.2% lower, the opposite to that observed for SMF samples in which it was 11.0% higher after 24 hr, compared with untreated samples. After 48 hr of fermentation in SSF and SMF, the vanillic acid content was 8.3% and 30.6% lower; however, after 72 hr of fermentation, it increased by 19.8% and 18.0%, respectively. *p*‐coumaric acid in fermented samples was reduced on average by 66.9%, compared with untreated samples. Ferulic acid in fermented samples was reduced on average by 40.3% in SSF samples and by 34.1% in SMF samples, compared with untreated samples. The sinapic acid content in fermented samples ranged from 7.68% (in 72 hr SSF samples) to 23.54% (in 72 hr SMF samples). The *p*‐hydroxybenzoic acid content increased in all fermented samples, compared with untreated samples (in 24 hr SSF and SMF samples by 45.1% and 52.4%, respectively; in 48 hr SSF and SMF samples by 35.5% and 38.0%, respectively; and in 72 hr SSF and SMF samples by 534.5% and 46.5%, respectively). In most of the BB samples tested, a decrease of 3,4‐hydroxybenzenecarboxylic acid was found, compared with untreated samples; the exception was 72 hr SMF samples, in which a 23.4% increase was established. Cereals are a good source of phenolic compounds, which include derivatives of benzoic and cinnamic acids, anthocyanidins, quinines, flavonols, chalcones, flavones, flavanones, and amino phenolic compounds. Although abundant in grains, many of these phenolic compounds are classed as bound since they are covalently linked with other cell wall constituents such as hemicelluloses that may limit their bioavailability (Wu et al., 2018). Solid state fermentation may assist in converting bound to free phenolics, thus increasing their bioavailability (Dey, Chakraborty, Jain, Sharma, & Kuhad, 2016). An increase in the bioavailability of phenolic compounds by lactic acid fermentation has been observed in rye, barley, oat and vegetables, and the dietary PA in flour from whole grain barley and oat groat is significantly improved after fermentation with LAB. Furthermore, it has been verified that ferulic acid can be transformed into 4‐vinylguaiacol, vanillic acid and trace quantities of vanillin by lactobacilli. Most likely due to the aforementioned biotransformation reactions, the ferulic acid content decreased by nearly 90% within the first 12 hr of fermentation. Kaur, Chakraborty, Kaur, and Kaur (2013) determined the ability of a panel of LAB isolates to release phenolics from agrowaste materials and biotransform them into industrially important compounds such as ferulic acid, 4‐ethyl phenol, vanillic acid, vanillin, and vanillyl alcohol. Additionally, *p*‐coumaric acid can be decarboxylated to the corresponding vinyl derivatives under the effect of a PA decarboxylase generated by LAB (Filannino, Cagno, & Gobbetti, 2018). However, bacterial cell growth is inhibited by hydroxytyrosol, oleuropein, tyrosol and vanillic, *p*‐hydroxybenzoic, sinapic, syringic, protocatechuic, and cinnamic acids at high concentrations (Dey et al., 2016). The increased bioaccessibility of *p*‐hydroxybenzoic and vanillic acids could be due to microbial enzymatic activity. Free *p*‐coumaric acid may be decarboxylated or reduced by *L. plantarum* PA decarboxylases or reductases to the corresponding phenol or vinyl derivatives. Therefore, these microbial metabolic pathways could explain the reduction of soluble *p*‐hydroxycinnamic compounds (Filannino et al., 2018).

**Table 4:** Phenolic acid (µg/g) content in untreated and fermented barley by‐products (BB)

Phenolic acid	Nonfermented samples	After 24 hr		After 48 hr		After 72 hr	
		SSF	SMF	SSF	SMF	SSF	SMF
Vanillic	7.85 ± 0.36^b^	6.11 ± 0.18^a^	8.71 ± 0.36^b^	7.20 ± 0.25^b^	5.45 ± 0.21^a^	9.43 ± 0.19^a^	9.26 ± 0.19^a^
*p*‐Coumaric	83.11 ± 0.28^b^	26.65 ± 0.23^b^	28.73 ± 0.28^b^	28.44 ± 0.36^c^	21.29 ± 0.63^c^	32.02 ± 0.62^c^	28.06 ± 0.65^b^
Ferulic	547.49 ± 0.61^c^	326.41 ± 1.51^e^	376.05 ± 0.31^b^	314.57 ± 3.81^d^	335.35 ± 3.61^d^	339.15 ± 3.87^e^	371.70 ± 4.36^e^
Sinapic	12.77 ± 0.43^c^	11.21 ± 0.40^b^	22.66 ± 0.43^c^	12.87 ± 0.52^c^	17.61 ± 0.69^d^	7.68 ± 0.85^c^	23.54 ± 1.20^c^
*p*‐Hydroxybenzoic	4.26 ± 0.37^b^	6.18 ± 0.47^b^	6.49 ± 0.27^a^	5.77 ± 0.25^a^	5.88 ± 0.28^a^	27.03 ± 1.20^e^	6.24 ± 0.69^c^
3.4‐Hydroxybenzenecarboxylic	6.45 ± 0.29^a^	3.73 ± 0.21^a^	3.03 ± 0.17^a^	4.16 ± 0.32^b^	2.93 ± 0.32^b^	5.48 ± 0.69^c^	7.96 ± 0.75^c^

Note

Data are the mean ± *SD* (*n* = 3).

Abbreviations: SMF, submerged fermentation; SSF, solid state fermentation.

^a–e^Mean values with different letters are significantly different when *p* ≤ .05.

### 3.5 Formation of BAs in barley by‐products during their treatment with *P. acidilactici* LUHS29

The BA content in BB samples is given in Table 5. Putrescine was the predominant BA in BB samples (in untreated samples, the putrescine content was 278.5 ± 4.3 mg/kg). Fermentation in all cases reduced the putrescine content in BB samples after 24 hr in SSF and SMF samples by 88.2% and 91.2%, respectively; after 48 hr in SSF and SMF samples by 92.4% and 93.7%, respectively; and after 72 hr in SSF and SMF samples by 71.4% and 97.9%, respectively, compared with untreated samples. The cadaverine content was also reduced during fermentation; compared with untreated samples, it was lower on average by 81.8% in fermented samples, and no cadaverine was determined in 72 hr SSF samples. Histamine was not determined in untreated or 72 hr SMF samples; however, in other fermented samples, the histamine content ranged from 0.9 ± 0.3 to 9.6 ± 1.0 mg/kg (in 48 hr SMF and 24 hr SSF samples, respectively). Tyramine was found in two of the seven samples analyzed in 24 hr SSF – 3.5 ± 1.3 mg/kg – and in 48 hr SSF – 4.5 ± 0.4 mg/kg. However, the highest total BA content was found in untreated samples (290.6 mg/kg), and fermentation reduced the total BA content after 24 hr in SSF and SMF samples by 84.2% and 90.9%, respectively; in 48 hr SSF and SMF samples by 88.8% and 93.1%, respectively; and in 72 hr SSF and SMF samples by 97.3% and 96.7%, respectively. It is known that histamine can be formed from histidine, tyramine from tyrosine, cadaverine from lysine, putrescine from arginine, and spermidine and spermine from arginine and methionine, and the toxicity of BAs depends on synergistic effects, for example, histamine toxicity is enhanced by the presence of cadaverine, putrescine, and tyramine (Renes et al., 2019). A moderate positive correlation was found between histamine and tyramine (*r* = .620), and a weak positive correlation was established between histamine and tyrosine (*r* = .176). A strong positive correlation was found between cadaverine and lysine (*r* = .727), a very strong positive correlation was established between putrescine and arginine (*r* = .937), and a moderate positive correlation was found between putrescine and methionine (*r* = .342). The results of the ANOVA test indicated a significant effect of fermentation conditions (SSF and SMF) and fermentation duration (*p* ≤ .0001), as well as interaction of these factors on the concentration of BAs in BB (for putrescine: *F*(302.143) = 2,295.855, *p* ≤ .0001; for cadaverine: *F*(8.035) = 7.335, *p* ≤ .005; for histamine: *F*(13.244) = 6.395, *p* ≤ .001; for tyramine: *F*(31.689) = 8.375, *p* ≤ .0001). Lactic acid bacteria have GRAS (Generally Regarded as Safe) status; however, they can be involved in BA accumulation in fermentable substrates. Finally, LAB are considered as the main BA producers in fermented foods. These compounds derive from AA decarboxylation through microbial activity and can cause toxic effects in humans, with symptoms (headache, heart palpitations, vomiting, and diarrhea) depending also on individual sensitivity (Barbieri, Montanari, Gardini, & Tabanelli, 2019). In particular, BAs can accumulate in fermented foods, and strains of lactobacilli, enterococci, lactococci, pediococci, streptococci, and leuconostocs have been associated with high levels of these compounds (Gardini et al., 2016). Histamine, tyramine, and cadaverine are associated with microbial contamination, principally bacterial, where *Pediococcus* and *Lactobacillus* are the main producer species (Almeida, Fernandes, & Cunha, 2012). Bas are harmful substances generated during the fermentation process. Regulations on the BA content in fermented foods are currently insufficient in comparison with the popularity of fermented food consumption (Park, Lee, & Mah, 2018). There are many reasons to prevent the accumulation of BAs in fermented products, mainly related to their utility as food quality indicators and their potential implications for consumer health. Controlling these compounds implies a deep understanding of the formation, monitoring and reduction of BAs during the processing and storage of food, and even of the effects of BAs in consumers after the digestion of foods containing different levels of these compounds (Ruiz‐Capillas & Herrero, 2019). The presence of BAs in a substrate is dependent both on contamination by decarboxylating microorganisms, on the availability of precursors (proteins and/or FAA), and on different intrinsic, environmental, and technological factors (Gardini et al., 2016). Reduction of BAs is often limited by the fermentation conditions; the main tool to counteract their accumulation is the choice of appropriate starter cultures able to rapidly and persistently colonize the fermentable substrate and which have the capacity to inhibit or reduce the growth of aminobiogenetic wild microorganisms (i.e., with strains showing bacteriocin production and/or antimicrobial properties) (Gardini et al., 2016).

**Table 5:** Biogenic amine content (mg/kg) in fermented and untreated barley by‐products (BB)

Biogenic amines	Nonfermented samples	After 24 hr		After 48 hr		After 72 hr	
		SSF	SMF	SSF	SMF	SSF	SMF
Putrescine	278.5 ± 4.3^b^	32.9 ± 1.7^a^	24.6 ± 2.1^b^	21.3 ± 1.9^b^	17.5 ± 1.9^b^	79.6 ± 4.3^c^	5.9 ± 1.3^c^
Cadaverine	12.1 ± 1.5^a^	2.4 ± 1.1^a^	1.9 ± 1.2^a^	1.3 ± 0.2^a^	1.7 ± 0.8^a^	–	3.7 ± 0.9^a^
Histamine	–	9.6 ± 1.0^a^	1.3 ± 0.7^a^	5.4 ± 0.6^a^	0.9 ± 0.3^a^	7.8 ± 1.2^b^	–
Tyramine	–	3.5 ± 1.3^a^	–	4.5 ± 0.4^a^	–	–	–
Total	290.6 ± 3.3^b^	46.0 ± 1.2^a^	26.4 ± 1.5^b^	32.5 ± 1.5^a^	20.1 ± 0.9^b^	87.4 ± 4.1^c^	9.6 ± 1.0^c^

Note

Data are the mean ± *SD* (*n* = 3).

Abbreviations: SMF, submerged fermentation; SSF, solid state fermentation.

^a–e^Mean values with different letters are significantly different when *p* ≤ .05.

### 3.6 Alkylresorcinol and lignan content in barley by‐products

**Figure 1:**
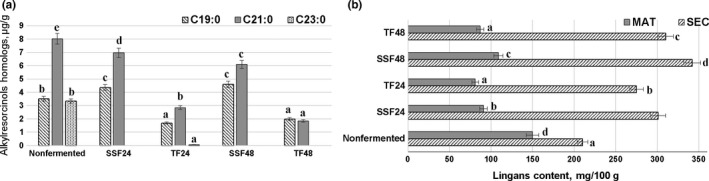
Alkylresorcinol homologue (µg/g) and lignan (mg/100 g) content in barley crop by‐products fermented with a *Pediococcus acidilactici* strain under submerged and solid state fermentation conditions

The alkylresorcinol (ARs) and lignan content in barley BB are presented in Figure 1a,b, respectively. Comparing the alkylresorcinol homologue concentration in BB samples, SSF increased the C19:0 content by 75.9% and 30.7% after 24 and 48 hr of fermentation, respectively; however, in SMF samples, the C19:0 content was 52.0% and 43.5% lower after 24 and 48 hr of fermentation, respectively. In all cases, the C21:0 content in fermented samples was lower, compared with untreated samples, and the greatest reduction was found in 48 hr SMF samples (reduced by 77.1%, compared with untreated). C23:0 homologue was not determined in most of the fermented samples (except in 24 hr SMF); however, in untreated samples, its content was 3.35 ± 0.03 µg/g. It has been reported that fermentation only changes the alkylresorcinol content slightly, and only minor changes are induced by LAB metabolism (Prückler et al., 2015). Our results are in agreement with Zhao et al. (2017) who explained that LAB produce acid in fermentation, and acidification leads to a decrease of ARs. Bran plays a key role in the overall health benefits of whole grains. Clinical trials and epidemiological studies have shown that the consumption of foods high in fiber is linked with a reduced risk of diseases such as colon cancer, diabetes, obesity, and cardiovascular disease, probably due to the phytochemicals (PAs, sterols, alkylresorcinols, vitamin E, and minerals) and fiber, which are embedded in the bran (Prückler et al., 2015). For this reason, collection of data about the changes of these compounds during technological processes is very important.

The lignan content in barley BB is presented in Figure 1b. It was found that the secoisolariciresinol (SEC) content was increased in all fermented samples after 24 hr in SSF and SMF by 43.3% and 31.0%, respectively, and in 48 hr SSF and SMF by 62.9% and 47.6%, respectively. However, opposite tendencies were established for the matairesinol (MAT) concentration in fermented samples, and in all the cases, MAT content in fermented samples was lower, compared with untreated samples after 24 hr in SSF and SMF by 39.3% and 46.0%, respectively, and in 48 hr SSF and SMF by 27.3% and 42.0%, respectively. Dietary lignans are of interest because they are associated with beneficial effects on human health; however, they have limited biological properties because of their low bioavailability and must be converted into enterolignans to exert their beneficial effects (Peirotén, Gaya, Álvarez, Bravo, & Landete, 2019). Jung et al. (2016) determined that fermentation increases the content of lignans, as well as their bioavailability. Our results are in agreement with Peirotén et al. (2019) who stated that fermentation increases the SEC content compared with control samples, and Jung et al. (2016) who determined that MAT is the least‐concentrated lignan in most foods studied, whereas SEC reaches the highest concentration.

## 4 CONCLUSIONS

Finally, both fermentation conditions reduced crude protein and crude fat content in BB; however, they increased dietary fiber content. Fermentation increased oleic, arachidic, eicosadienoic, behenic, and lignoceric FA in BB samples. Comparing PAs, vanillic acid was increased after 72 hr of SSF and SMF (18.9%). Also, *p*‐hydroxybenzoic acid was increased by prolonging the fermentation time (after 72 hr of SSF and SMF by 534.5% and 46.5%, respectively). The predominant BA in BB was putrescine, and fermentation with *P. acidilactici* strain LUHS29 reduced the total BA content in BB. To get higher concentrations of alkylresorcinol homologue C19:0, SSF should be used, as after 24 hr of fermentation the C19:0 content increased by 75.9%. The SEC content during fermentation was increased in all the fermented samples (on average by 46.2%); however, the MAT content in fermented samples was lower, compared with untreated samples. Finally, the results of this study can be used for recommendations according to valorization of by‐products and their further use in food, pharmaceutical or feed industries.

## CONFLICT OF INTEREST

The authors declare no conflict of interest.

## ETHICAL APPROVAL

Human and animal studies were not included in this experiment.
